# Carer and staff perspectives on supplementary suckling for treating infant malnutrition: qualitative findings from Malawi

**DOI:** 10.1111/mcn.12064

**Published:** 2013-06-25

**Authors:** Natasha Lelijveld, Chawanangwa Mahebere‐Chirambo, Marko Kerac

**Affiliations:** ^1^ UCL Centre for Global Health University College London London UK; ^2^ College of Medicine Blantyre Malawi; ^3^ Leonard Cheshire Disability and Inclusive Development Centre, Department of Epidemiology and Public Health University College London London UK

**Keywords:** breastfeeding, infant and child nutrition, qualitative methods, infant feeding, breastfeeding support, lactation, supplementary suckling

## Abstract

Severe acute malnutrition (SAM) in infants aged <6 months is a major global health problem. Supplementary suckling (SS) is widely recommended as an inpatient treatment technique for infant <6 months SAM. Its aim is to re‐establish effective exclusive breastfeeding. Despite widespread support in guidelines, research suggests that field use of SS is limited in many settings. In this study, we aimed therefore to describe and understand the barriers and facilitating factors to SS as a treatment technique for infant SAM. We conducted qualitative interviews and focus group discussions in a hospital setting in Blantyre, Malawi, with ward staff and caregivers of infants <2 years. We created a conceptual framework based on five major themes identified from the data: (1) motivation; (2) breastfeeding views; (3) practicalities; (4) understanding; and (5) perceptions of hospital‐based medicine. Within each major theme, more setting‐specific subthemes can also be developed. Other health facilities considering SS roll‐out could consider their own barriers and facilitators using our framework; this will facilitate the implementation of SS, improve staff confidence and therefore give SS a better chance of success. Used to shape and guide discussions and inform action plans for implementing SS, the framework has the potential to facilitate SS roll‐out in settings other than Malawi, where this study was conducted. We hope that it will help pave the way to more widespread SS, more research into its use and effectiveness, and a stronger evidence‐base on malnutrition in infants aged <6 months.

## Introduction

Reducing malnutrition in all its forms is a key to reducing poverty and achieving important global health targets, such as the Millennium Development Goals (Gillespie & Haddad 2003). Child malnutrition accounts for 35% of child disease burden and 11% of total global disability‐adjusted life years (DALYs) (Black *et al*. 2008). Severe acute malnutrition (SAM) has a particularly high case fatality rate (Schofield & Ashworth 1996; Heikens 2007), and its treatment has received much recent international attention (WHO/UNICEF 2007; ENN 2011). Ready‐to‐use therapeutic foods (RUTF) have become particularly important because they do not need preparation, can be given by carers at home and thus enable outpatient care of SAM. This has far greater potential for public health impact than previous inpatient‐focused approaches (Collins *et al*. 2006). One ‘side effect’ of this success with RUTF is, however, a lack of attention on SAM in infants aged under 6 months (infants <6 months). As their target diet is exclusive breastfeeding (EBF), infants <6 months are ineligible for RUTF and are more complex and time‐consuming to manage. As a result, they are often side‐lined in SAM treatment programmes (ENN/UCL/ACF 2010). This neglect is slowly changing. Dispelling a common assumption that SAM is rare in this age group, one recent study estimated that of 20 million children under 5 years with SAM worldwide, 3.8 million are infants <6 months (Kerac *et al*. 2011). Forthcoming World Health Organization (WHO) guidelines on SAM will, for the first time, include a dedicated section on infants <6 months (WHO 2012b). With this important step forward, it is important that treatment of infant <6 months SAM is informed by a solid evidence‐base. Unfortunately, this is currently lacking.

In a recent review of 36 SAM guidelines from countries in Africa, Asia and South America, 29 had sections on infants <6 months. Of these, 28/29 (97%) recommended supplementary suckling (SS) as the core treatment for this patient group (Kerac *et al*. 2012). The aim of SS (also sometimes called supplemental suckling; nursing supplementer, LactAid supplementer or breastfeeding supplementer) is to establish or re‐establish effective exclusive breastfeeding. It implicitly recognises the unique composition of breast milk and its irreplaceable role in infant nutrition (Hanson *et al*. 2009; Berry & Gribble 2008). Briefly, SS involves taping a narrow nasogastric tube (NGT) to the breast so that one end is next to the nipple and the other end is in a cup of modified therapeutic milk, encouraging the infant to suckle at the breast so as to stimulate breast milk production; as the infant is suckling, simultaneously giving specially formulated therapeutic milk via the tube. As breast milk production increases and the infant's weight begins to improve, the modified therapeutic milk top‐ups are gradually reduced; finally, the infant is discharged home on EBF alone.

Despite being widely recommended in national SAM guidelines, SS use in practice is difficult to determine. One chapter of the recent MAMI report (Management of Acute Malnutrition in Infants; ENN/UCL/ACF 2010) described field experiences with infants <6 months. Key informants, mostly from non‐governmental agencies working in SAM‐prevalent developing country settings, were interviewed. They often mentioned SS but opinions were sharply divided: some respondents reported great success; others felt it largely ineffective and seldom used it. One interpretation of these differences is that SS is efficacious but difficult to implement, especially without the right on‐site support and expertise. Identifying key barriers and facilitators to SS is therefore important and will become even more so when the new WHO infant SAM guidelines are released (recommending SS for infant <6 months inpatient care) (WHO 2012b).

In this study, our objectives were to address this evidence gap and describe carer and staff perspectives on barriers and facilitators to SS in a large district/referral hospital in Malawi, Africa. As well as informing practice improvements in this setting, our aim was also to share this experience by developing a framework, which could be adapted and used by others to explore their own, locally applicable, barriers and facilitators.

### Key messages

- Supplementary suckling (SS) is widely recommended for treating severe acute malnutrition in infants aged <6 months. However, implementation is not easy and use is thus limited.
- Better understanding of the barriers and facilitating factors to SS is important to enable more widespread field use. We sought perspectives on this from both caregivers and health care staff in Malawi.
- We identified five themes for guiding discussions when programmes or health facilities are implementing SS: motivation, breastfeeding views, understanding, practicality and perceptions of hospital‐based medicine. A framework based on these themes can be used to create a context‐specific action plan for effective SS.

## Materials and methods

### Study design and theoretical framework

This was a qualitative study, consisting of semi‐structured interviews and small focus group discussions (FGDs). Content analysis theory underpinned the study design, with elements of action research theory also playing a role, as participants were encouraged to identify problems and solutions within the scope of the topic (Hult & Lennung 1980; Graneheim & Lundman 2004). This report follows COREQ reporting guidelines (Tong *et al*. 2007).

### Setting and participant selection

The study was conducted at the Queen Elizabeth (QE) Hospital in Blantyre, Malawi, in June and July 2012. This is a large teaching/referral hospital, which also serves as the district hospital for urban and rural Blantyre. Health care staff and carers were recruited from the malnutrition ward (which accepts infants and children of all ages for treatment of SAM) and the nursery ward (which accepts infants aged <6 months admitted from the community, some of whom may be malnourished). Typical of many other settings (ENN/UCL/ACF 2010), neither ward routinely uses SS despite it being recommended in Malawi national guidelines, and infants <6 months are not routinely screened for SAM.

Two groups were recruited into the study:

1. Health care staff at QE hospital: Convenience sampling was used to recruit nurses and health attendants to participate in semi‐structured interviews. These are the cadre who would be responsible for SS implementation. They were recruited by direct face‐to‐face approach with the help and permission of lead clinicians from the respective wards.
2. Mothers/Carers: Participants had to be the mother or female carer of infants aged <2 years, either breastfeeding or non‐breastfeeding, achieved through purposive sampling (Patton 1987). The cut‐off of 2 years was chosen because WHO recommends that breastfeeding should still occur up until this age, and SS, although focused on young infants, is still relevant beyond the first 6 months. Eligible participants were identified and approached face‐to‐face with the help of ward nurses. The nurses explained the study to potential participants and asked whether or not they would participate.

**Figure 1:**
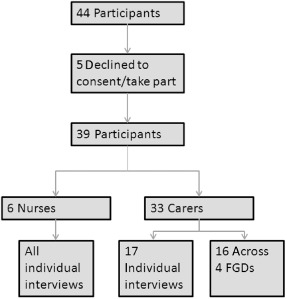
Flow diagram depicting sampling and sample size.

Twenty‐two semi‐structured interviews and four FGDs were conducted, made up of 6 nursing staff and 17 carers. They took place in quiet rooms near to the wards; only NL, the research assistant (CC), the participant and the participant's child were present. Data collection continued until saturation was reached (see Fig. 1 for resultant sample size and details of dropouts). Reasons for dropout/not taking part were not given.

### Data collection

Interviews and FGDs all began with a standardised explanation of SS. This was developed during a pilot phase of the project, and included an image of SS in action, taken from Malawi national malnutrition guidelines. Participants were then encouraged to ask any questions. If requested, further clarifications were provided.

Topic guides were designed to be open‐ended, sensitive, clear and would encourage participants to share as much relevant information as possible, in their own words (Patton 1987). Following the description of SS and questions, participants were asked their views of breastfeeding and what they think of SS as a treatment technique from SAM in infants. Additional prompts regarding the good things and bad things about the treatment, whether Malawian women would be happy to use it, whether they themselves would be happy to use it if they were in the relevant situation and suggestions to improve the technique were also asked when necessary. The topic guides were initially designed by NL and MK, and further refined after presentation at an expert meeting on infant feeding in March 2012 (ENN 2012).

The interview topic guide was piloted on three mothers and one nurse; the FGD topic guide was piloted on one focus group; both were then discussed with the research assistant (CC) prior to completion. All participants were offered a convenient time and use of a translator (done by research assistant, CC). No staff members requested translation; all but one of the mothers did. All participants were female and ages ranged from 17 to 45. Only one interview/FGD was done for each participant. Interviews lasted between 20 and 40 min; FGDs lasted between 40 and 75 min. All of the carers had breastfed their child to some extent but only some were still breastfeeding at the time of interview. None of the participants had ever used SS or seen SS being used.

Data were recorded on a digital recording device (OLYMPUS DM‐650, Olympus, Beijing, China). It was then simultaneously translated and transcribed by CC to produce English transcripts. Translation was verified during pilot phase by an independent researcher and no major discrepancies were found. NL took field notes both during and after interviews/FGDs. FGDs consisted of four or five participants as it was not feasible to gather more participants in a busy hospital setting. Also for this reason, FGDs only took place with mothers/carers, not with nursing staff.

### Data analysis

Throughout data collection, NL read the transcripts and developed labels representing recurring themes (‘codes’). This was done deductively from the interview guides, e.g. ‘good things about SS’ and ‘hygiene concerns’, and inductively using content analysis, e.g. ‘concerns for child's comfort’ and ‘dislike of intervention’ (Denzin & Lincoln 2005). This iterative process resulted in some additions to the topic guides during the data collection period. Data collection continued until saturation was reached, as defined by Morse (1995), i.e. NL and CC felt that the new data being collected were no longer creating any new labels or ‘codes’. As key themes began to emerge, they could begin to be grouped and merged. Subsequent analysis involved joint reading by NL and the research assistant (CC) followed by long‐table method to further link, expand and refine themes, until the final results were created (Krueger & Casey 2000). For example, ‘hygiene concerns’, ‘time pressures’ and ‘responsibility’ were grouped under ‘practicalities’. Transcripts were printed, highlighted, cut and grouped; no computer software was used. CC's active role as an additional coder was important due to his prominence during the interviewing and translating process.

### Research team and reflexivity

The lead researcher (NL) is of a different nationality and ethnic origin to participants but is of the same gender. An MSc student at the time, she was introduced as ‘a student researcher’ in an attempt to reduce the hierarchy between researcher and interviewees (DiCicco‐Bloom and Crabtree 2006). The motivation for the study was explained as ‘looking to improve treatment options for malnourished babies in Malawi’. NL built rapport with participants by spending time on the wards prior to and during the study. This was especially valuable for nurses who were sometimes wary of participating. Younger mothers were also shy to participate. As their interviews were all in the local language, Chichewa, the mothers communicated largely with CC (research assistant) who is male but of the same ethnic origin and culture as participants. CC has a Social Science BSc and several years' experience conducting qualitative research.

### Ethics

Ethical approval was granted by both Malawi College of Medicine Research Ethics Committee, COMREC (reference P.03/12/1187) and UCL Research Ethics Committee (reference 3679/001) prior to commencement.

## Results and discussion

Five ‘major themes’ emerged, which relate to each of the barriers, facilitating factors and solutions discussed by the participants. These were motivation, breastfeeding views, practicality, understanding and perception of hospital‐based medicine. They are discussed in more detail below. We believe that they are broad enough to be applicable to other programmes and settings considering SS; however, this is yet to be tested. Subthemes may be less generalisable but should certainly be considered as a guide to possible local issues elsewhere:

### Motivation

Informants described how motivation of mothers, carers and nurses can affect SS both positively and negatively. They described how the success of SS may depend on the level of ‘interest’ the mother has in using it and how hard she is willing to persevere. Motivation can also be affected by concerns for the infant's comfort, apprehension that the infant might not take to the breast having stopped breastfeeding and the idea that the mother will do whatever is best for the infant. This latter sentiment was expressed many times and was a strong motivator in the decision as to whether a mother could ‘accept’ SS or not.

I could use it for I want to rescue the life of the child. Aren't you wishing the child just good? (Mother on the Nursery Ward)

It doesn't look difficult, though maybe for a child who has stopped breastfeeding, for the first time I don't know if he will respond to the breast. (Mother on Malnutrition Ward)

### Breastfeeding views

Carers' views about breastfeeding are clearly critical to SS as EBF is the ultimate aim of the technique. The general consensus was that all mothers want to breastfeed their children; surprise was expressed at even being asked this question. This positive attitude towards breastfeeding in Malawi is a great facilitating factor towards SS. The majority response that the technique looked ‘easy’ may also arise from this strong culture of breastfeeding. It is important to remember, however, that these relaxed opinions of SS were expressed following an in‐depth explanation of the technique and with ample time for questions.

All mothers want to breastfeed their children, that is our culture, we breastfeed up until 6 months. But the problem is exclusive breastfeeding, I think that's where the problem is. All mothers breastfeed their children and they want to breastfeed their children. (Nurse on Malnutrition Ward)

Siyovuta – It is not a difficult one. (Mother during FGD)

Many caregivers expressed a good understanding of the benefits of breastfeeding, including detailed aspects, such as EBF, protective properties of breast milk and the benefits of colostrum. This evidence that complex breastfeeding messages have been well received is arguably in conflict with the high number of malnourished infants thought to be present at the hospital. This probably reflects the fact that knowledge does not automatically lead to changes in EBF behaviours.

It is a very good technique, for even the nurses tell us that essential milk a child requires to be healthy comes from the breast, so I find it a very good one. It is because the first breast milk is very essential to a young child, then it is a good technique so that you breastfeed from the time the child has been born. (2 Mothers from Malnutrition Ward during a FGD)

Mothers who choose not to breastfeed present a challenge for implementing SS as they likely do not want to re‐establish EBF. Reasons suggested for not breastfeeding included changes of physical appearance, working mothers and not wanting to smell of milk. The most common reason, however, was HIV infection (‘matenda amasiku anowa’ – the nowadays disease). Mothers with HIV are currently encouraged to breastfeed their infants exclusively for 6 months as the default option (WHO 2009). This contrasts previous guidelines, which were more open to formula feeding as the starting point for feeding decisions. Participants' responses suggested that this guideline change has confused caregivers. In many cases, the revised message has not been received, many fearing that it is dangerous to breastfeed if HIV+.

If she is HIV infected then it is not right for her to breastfeed the child. (Mother in FGD)

CC: ‘why do some mothers choose not to breastfeed?’ ‘Because of this so common disease *[HIV]*. They just feel now, when I am reactive I am going to infect my baby, so instead I shouldn't just feed. Yet in the hospital they are still told can feed up to 6 months, then after 6 months they shouldn't breastfeed. But some they go to the private hospital but the counselling is somehow different to the counselling that we do here’. (Nurse on Nursery Ward)

In future SS guidelines, such messaging around HIV will be critical. A key question at present is: can HIV+ carers use SS? And if not, what should be offered instead? Mixed feeding, an inherent part of SS, is associated with the highest risk of HIV transmission (WHO 2007); however, studies have not yet compared this risk against that of not receiving SS and not re‐establishing EBF.

### Practicality

When participants were asked for their thoughts on ‘hygiene, cost, comfort and time’, most participants focused on hygiene issues. They felt that this was important and more complicated for SS because the tube is more difficult to clean than a cup or spoon, and it is necessary to bath several times a day if breastfeeding. They did, however, feel that such obstacles could be overcome. In QE hospital, NGTs are commonly used for enteral feeding and mothers are responsible for ensuring that they remain clean; caregivers felt that the same practice could apply to SS.

Hygienically I don't know how I can keep it clean because milk will be going inside there and maybe the milk will clog it. So you have to find ways to clean it. (Mother on Malnutrition ward)

I think first of all, the mothers should have a health education about the tube, yes, how to take care of it. (Nurse from the Malnutrition Ward)

Both staff and carers were concerned about the time it would take to make up the milk and attach the tube, especially if the baby is crying. As a solution, informants suggested that extra staff be recruited specifically to manage the practicalities and support required for SS, current nurses being already stretched beyond capacity.

‘It could be difficult, the baby is crying and you have to take a pipe and plaster it to your breast’. ‘No I cannot use it because I could have difficulties in preparation. I cannot manage’ (2 Mothers from Nursery ward during FGD).

‘Maybe there should be somebody to help them because a child could be crying and needs to breastfeed …’ (Mother on the Nursery Ward)

### Understanding

Many perceived barriers to SS arose through poor understanding of certain aspects of the technique. This theme generated the most data and was highly multifaceted. Participant responses were generally short, perhaps due to the novelty of the topic, with the majority of discussion arising around questions about the technique and in explaining their own understanding of the technique to one another during FGDs. This fact is quite telling in itself. Encouragingly, most barriers discussed are simply solved by explanation and education. Many participants expressed the view that they would be happy to use SS because they have understood it and because it had been explained in detail; however, they expressed doubt about other mothers wanting to use it because they may not understand. Aspects that caused the greatest confusion included how the tube is used, risks to the mother, how SS relates to HIV, how to source the therapeutic milk and general worries about the technique being new and unknown. The NGT has a bad reputation among Malawian women; it is associated with a very sick child who is more likely to die, an association commonly confused with causality. Using NGTs for SS could thus be a barrier to its implementation in this setting. Generally, however, when mothers understood that the tube was not inserted into the child's body, they were happy to accept it. Other confusions included fears that the tube would pierce the breast or would harm the mother, such as causing cancer.

The problem is telling others of this technique; they could be scared that you would pierce through their breast with the tube or feed the infant through the nostrils. (Mother on Malnutrition Ward)

Because when new things are introduced others find it difficult to understand, many could say they initiate cancer for example, they say they could cause diseases. But we have properly understood, you have explained to us in detail. (Mother in FGD)

Mothers have a feeling that nasogastric tubes kill their children because they go inside and into the intestines. (Nurse on Malnutrition Ward)

Many participants worried about sourcing materials (tube and milk) when at their homes or in the community, despite explanations that SS is only for hospital use and not for continuation after discharge home, this being conditional on EBF resumption. This suggests that the physiological demand/supply mechanism underlying breastfeeding was not readily understood. Many felt that it is the act of giving birth or the flow of blood, or a woman's age that influence lactation rather than stimulation.

Now when one has been discharged and is going home, are you going to provide the tube to be using? (Mother from the Nursery Ward during a FGD)

### Perceptions of hospital‐based medicine

Perceptions of hospital‐based Western approaches to medicine (in contrast to traditional medical perspectives, which are also very influential in Malawi) can both facilitate and limit the implementation of SS. In Malawi, we found that SS could be negatively perceived as it was considered interfering in natural occurrences and in conflict with fatalistic beliefs.

Now no, I would not use it. Let God be God. (Mother on the Malnutrition Ward)

In addition, hospitals are viewed as dangerous places, the obvious association with frequent death again being confused with the hospital causing death. Other perceptions facilitated SS: the culture of listening to the doctor once you have committed to going to hospital, and a view that SS is better than some of the alternative feeding methods. Several participants said that they would do whatever the doctor advised, some explained that they had been discouraged from coming to the hospital by their community, but had persevered. This culture of complying with doctors' orders may facilitate the implementation of SS; however, it could also be a barrier if it means that mothers do not voice their concerns about the technique. A mother's own confidence in the technique is thought to affect SS, just as it affects breastfeeding. Hence, it is important that mothers ask questions to ensure self‐efficacy with the technique and a greater chance of success (Bandura 1977).

I say whatever the medical personnel advise me, I will follow that. (Mother on Nursery Ward)

When I came here [the hospital] I saw that all those problems are gone. He is able to eat properly. Then I learnt that when the doctors are speaking it is better to follow. (Mother on Nursery Ward)

**Figure 2:**
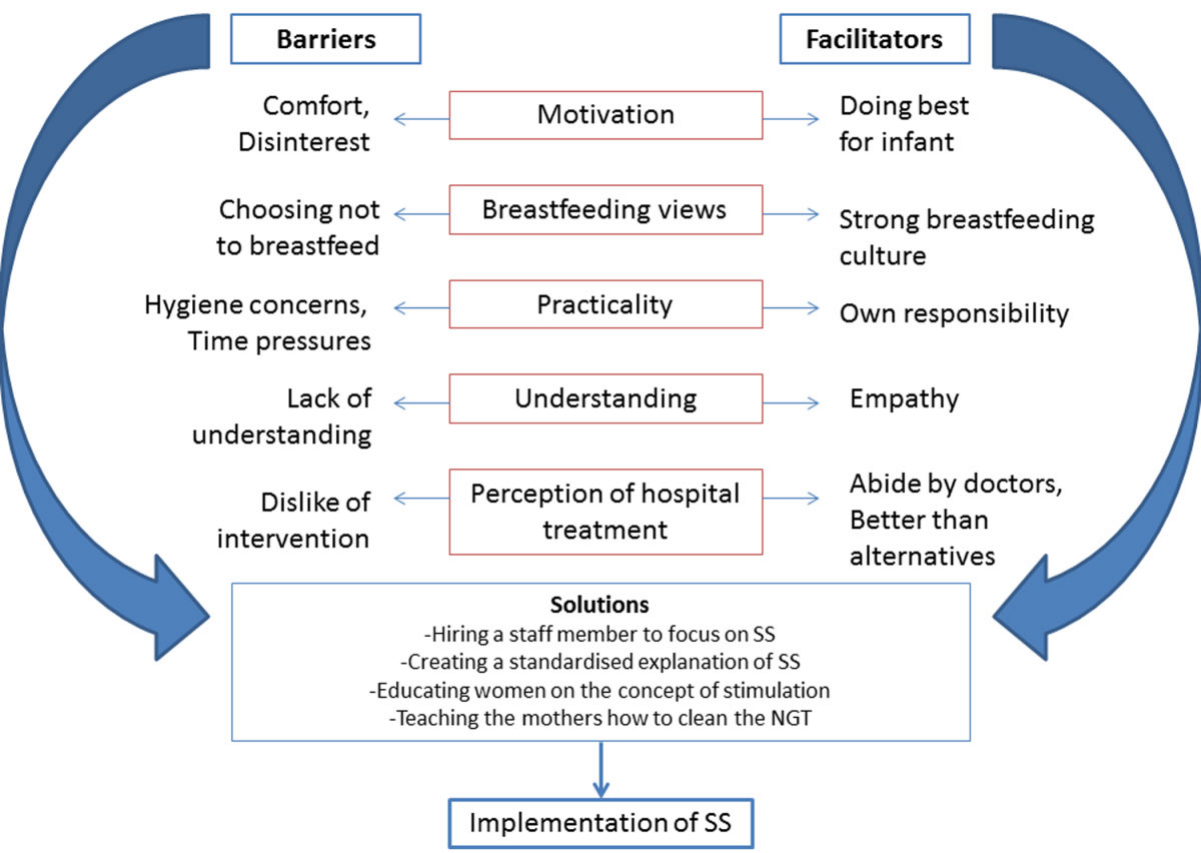
Framework outlining the barriers, facilitators and solutions identified in the Malawi setting within the five major themes. NGT, nasogastric tube; SS, supplementary suckling.

Having identified these potential barriers and facilitators, it is important to consider how each facilitator can be maximised and each barrier overcome. Figure 2 shows a suggested solutions‐framework based on data from our study. This is most directly relevant to SS at QE hospital where this study was done. However, using the five major themes as a foundation, we believe that minor local adaptations would make a similar version of the framework useful elsewhere too. In settings that are similar to QE or Malawi, such adaptations might be very quick and easy for local experts to do without the need for extensive consultation. In very different contexts, wider consultation with focus groups or interviews would add value and ensure local applicability.

Specific actions for our Malawi context, arising from our data and the resulting framework below, include hiring an extra member of staff to focus on SS/infant <6 months SAM alone. This addresses several barriers in one. ‘Motivation’ e.g. is affected by the level of staff support received (Oberlin & Wilkinson 2008). Staff support can also alleviate ‘understanding’ barriers by allowing adequate time for explanations. Indeed, guidelines often stress the need to ‘support’ the mother, but what exactly this entails and how long it takes is never detailed. Providing information and adequately addressing maternal/carer concerns is another key action. Based on our data, we suggest answers to ‘frequently asked questions’ (FAQs) in Table 1 for use by ward staff explaining SS to potential recipients. Again, these may need some adaptation for use in other settings and contexts, but in all cases offer an important starting point (see http://onlinelibrary.wiley.com/doi/10.1111/mcn.12064/suppinfo for additional participant quotes).

**Table 1:** Frequently asked questions about supplementary suckling (SS), which arose during data collection and model responses

How will I source milk/the tube when I am at home?	You won't need to! SS is only needed for several days in hospital at the beginning of treatment. It will help both your infant recover by getting enough milk AND will help you produce more breast milk/restart breastfeeding if you've previously stopped. As you produce more breast milk, the amount of therapeutic milk given to your infant will be gradually decreased. It will eventually be stopped altogether once your infant has improved and is growing well again.
Does the tube go inside the infant? Does the tube pierce the mother's breast?	No! The tube is gently taped on top of the breast at the nipple. The infant puts both the tube and the nipple in their mouth and sucks. The tube does not pierce the breast or go inside the infant beyond the lips.
How will I pay for the milk?	The hospital will supply you with the milk needed for treatment. When the treatment is finished, you will be able to breastfeed and will therefore not need to buy milk at home.
I cannot use this because I am not producing milk.	This is a common misunderstanding. Rather than milk coming and then the infant sucking, it is the baby suckling at the nipple which stimulates your body to produce more breast milk. The more he/she sucks, the more milk will come. So, even if you are not producing milk at the beginning of the treatment, you will start producing milk as the days go on. Your infant can suckle at the nipple to start the milk flow but they will not be frustrated because they will take formula milk until your breast milk flows.
Is this technique used in other places?	Yes, it is widely recommended by feeding and health experts and is used in many countries, rich and poor, around the world.
Is the formula milk safe for my baby?	The formula milk used for SS is for short‐term use only. Properly and freshly (never use old milk that's been left lying around!) prepared by your hospital it is safe but it does not replace breast milk, as the only normal and safe food for your infant. This is why the aim of SS is to restart breastfeeding as soon as possible.

## Study limitations

As already highlighted, generalisability of our findings is the biggest potential limitation of our work being set in one site alone. To overcome the problem, we deliberately made the five major themes in our framework as broad as possible. Hence, even if our respondents had particular individual biases in terms of how they perceived SS, the *theme heading* should still be valid for others who see things differently or come from a very different cultural/social/economic background. In addition, none of the women in the study had ever seen or used SS, so their answers were based on theory rather than practice; their views may well be different after having used the technique (this is, however, a situation which many other settings trying to start SS for the first time will be in).

Another limitation was that repeat interviews were not carried out and participant checking did not occur due to logistical difficulties as well as time and budget constraints. Given consistency of messages and the fact that saturation was reached in sampling, we did not feel that this represents a major impediment to our results.

Other limitations of the study are linked to language. In this research, the lead researcher (NL) was only able to read translated transcripts rather than listen to the direct source of the quotes, thus, potentially losing assumptions, feelings and hidden values carried by spoken language (Phillips 1960). It was to capture some of these data and understand differences of concepts across languages that CC acted as both researcher and translator, taking part in discussions about his interpretations of participant's views and in the analysis and coding process. CC's experience and presence throughout the study allowed for consistency during the data collection process and improved conceptual congruency in the overall translation process (Twinn 2008). Finally, researcher's characteristics and background may have affected responses; NL was a woman but of different social and ethnic origin to respondents, this may have created barriers (Britten 1995). CC, in contrast, was from the same culture and understood it well, greatly enhancing data richness. But he was also a man. This may have affected what female respondents were comfortable with saying.

## Conclusions

Suboptimum breastfeeding, particularly non‐EBF in the first 6 months of life, accounts for approximately 10% of the disease burden in children under 5 months (Black *et al*. 2008). EBF has important long‐term as well as short‐term benefits (WHO 2012a); and the consequences of undernutrition in infancy are well documented and have recently received considerable public attention as a result of the ‘Scaling Up Nutrition’ (SUN) movement (UNSCN 2010). This emphasises the first ‘1000 days’ from conception to 2 years when nutrition can have lasting impacts on health, brain development, intelligence, educability and productivity (SUN 2010) (Victora *et al*. 2008). There is, in short, little doubt that re‐establishing effective EBF with SS is a potentially vital child health intervention. Its effectiveness in poor‐resource settings deserves to be taken seriously. While shown to work well in some settings (Corbett 2000; Oberlin & Wilkinson 2008), wider evidence is very limited and there are anecdotal suggestions that implementation is difficult. In this study, we identified relevant barriers and facilitators. Using a bottom‐up approach to understand the views of mothers and relevant staff, we found that perspectives could be classed within five major themes: motivation, breastfeeding views, practicality, understanding and perceptions of hospital‐based medicine. Used to shape and guide discussions about SS and inform action plans for implementing SS, we believe that this framework has potential to facilitate more successful SS roll‐out in settings other than Malawi, where this study occurred. We hope that it will pave the way to more widespread SS, more research into its use and effectiveness, and a stronger evidence‐base on infant <6 months SAM.

## Sources of funding

This work was self‐funded.

## Conflicts of interest

The authors declare that they have no conflicts of interest.

## Contributions

MK largely conceptualised this research, while NL and CC conducted the data collection and analysis. All authors were responsible for interpretation of findings and writing of this paper.

## Supporting information


**Appendix S1.** Additional quotes.
