# Supplementary material for: Carer and staff perspectives on supplementary suckling for treating infant malnutrition: qualitative findings from Malawi
Source: Matern Child Nutr. 2013 Jun 25;10(4):593–603. doi: 10.1111/mcn.12064 (PMC6860316; doi:10.1111/mcn.12064)
Supplement: Supplementary file 1 — Appendix S1. Additional quotes. [file MCN-10-593-s001.docx]

**Additional Quotes:**

**Motivation:**

*“If you have interest and you can see its advantage, you can accept it”* Mother on malnutrition ward

*“Suckling would manage when you insist so much the child could manage but if you are lazy the child will not be able to feed”* Mother on malnutrition ward

*“Maybe the challenging part on the combination of the nipple and the tube, won’t the child find it a mouthful?”* Mother on nursery ward

**Breastfeeding Views:**

*“My thoughts are that the technique is good because if you are sick and you are not eating you can prepare the milk for the child and place the tube of the breast and will be suckling from there”* Mother on malnutrition ward

CC: “Why do some women choose not to breastfeed?”

*“Some are working so that they are afraid that the child will be missing the mother, so it is better not to breastfeed”* Mother on nursery ward

*“Maybe they know that they don’t have enough body food, then they buy milk”* Mother on nursery ward

**Practicality**

*“It is good that before connecting the tube on your breast to feed the baby you should take a bath and also you should make sure that the cup you use for the milk is washed”* Mother on malnutrition ward

*“Somehow I think it* [SS] *could not be good because the milk there is in a cup, it is easy for germs to go inside”* Mother on nursery ward

*“There on the cup, hygiene will be difficult; it requires you to finish the milk. If you prepare a lot of milk and it has remained could it be possible to keep it?”* Mother in FGD

*“They will see it as time-wasting, using the tube than using a cup and spoon”* Nurse on nursery ward

*“There is nothing that could discourage somebody... there is not a task that could tire you as compared to what she does at home”* Mother on malnutrition ward

*“I don’t think you can say it’s expensive because this is an ordinary cup and these are the ordinary tubes, so I don’t think it’s too costly. Yeah I think we can manage this locally”* Nurse on nursery ward.

**Understanding:**

*“Some would not be happy to use it because of not understanding how the technique is working”* Mother on malnutrition ward

*“Because it will be like a new thing they have never seen it, for them this thing is new so you know ‘attitude’. Attitude to change, its difficult most of the time”* Nurse on malnutrition ward

*“There are some who could ask but others won’t, they will just start telling others that I have found such-such a mother feeding a child with a tube, I think she is HIV infected… they will say you have a problem... saying she is just pretending to use a breast but the main feeding channel is the tube”* Mother in FGDs

*“Also I don’t know how much is the milk, or what type or who will be offing it. Would we be buying?”* Mother on nursery ward

*“I can use it for my breasts take time to start producing milk… they even take 2 weeks after giving birth before starting lactating”* Mother in FGDs

*“So if a child has been left by a mother and someone else takes it, it is difficult for the breast to start lactating, it is difficult, others even take a month before the milk production has started”* Mother in FGDs

**Perceptions of Hospital Treatments:**

*“It is easy because you are not inserting the tube in the throat; if they are putting in the throat as they do with the nostril tubes then I would have said not good. But you are saying it is just plastered on the breast then I think it is a very good way of helping”* Mother on malnutrition ward

*“If the child is still young you might not be able to put a spoon in his mouth or some also from the ‘plastic breast’* [bottle] *they just spit, don’t feed, while this one will think is feeding from the breast. Then all things will be fine”* Mother on nursery ward
